# Ecological Fall Prediction Sensitivity, Specificity, and Accuracy in Patients with Mild Cognitive Impairment at a High Risk of Falls

**DOI:** 10.3390/s23156977

**Published:** 2023-08-06

**Authors:** Chaesu Kim, Haeun Park, Joshua (Sung) You

**Affiliations:** 1 Sports Movement Artificial-Intelligence Robotics Technology (SMART) Institute, Department of Physical Therapy, Yonsei University, Wonju 26493, Republic of Korea; kar3959000@naver.com (C.K.); evepark16@yonsei.ac.kr (H.P.); 2 Department of Physical Therapy, Yonsei University, Wonju 26943, Republic of Korea

**Keywords:** mild cognitive impairment, sensitivity, specificity, accuracy, postural sway, fall, ecological environments

## Abstract

While falls among patients with mild cognitive impairment (MCI) have been closely associated with an increased postural sway during ecological activities of daily living, there is a dearth of postural sway detection (PSD) research in ecological environments. The present study aimed to investigate the fall sensitivity, specificity, and accuracy of our PSD system. Forty healthy young and older adults with MCI at a high risk of falls underwent the sensitivity, specificity, and accuracy tests for PSD by simultaneously recording the Berg Balance Scale and Timed Up and Go in ecological environments, and the data were analyzed using the receiver operating characteristic curve and area under the curve. The fall prediction sensitivity ranged from 0.82 to 0.99, specificity ranged from 0.69 to 0.90, and accuracy ranged from 0.53 to 0.81. The PSD system’s fall prediction sensitivity, specificity, and accuracy data suggest a reasonable discriminative capacity for distinguishing between fallers and non-fallers as well as predicting falls in older adults with MCI in ecological testing environments.

## 1. Introduction

In 2021, the World Health Organization reported that, worldwide, approximately 20 million (or one-third) of community-dwelling older adults with mild cognitive impairment (MCI) over 65 years of age experience one or more falls each year [1]. Falls among patients with MCI have been associated with increased postural sway during ecological activities of daily living, including sitting, standing, and walking [2]. Recent evidence suggests that altered dynamic balance performance was observed in 15.1% and 18.2% of older adults with MCI or mild or moderate dementia at a high risk of falls using the Timed Up and Go (TUG) test ([3] and Berg Balance Scale (BBS), respectively [4], when performed with no or minimal contextual interference or in a laboratory or controlled closed environment. In contrast, abnormal dynamic balance performance was noted during TUG [5] and BBS [6] testing in 36.3% and 21.3% of older adults with MCI or mild or moderate dementia, respectively, when performed in a substantially compromised or ecological community environment with higher contextual interference (e.g., dual cognitive motor tasks, divided attention tasks) [7,8]. This confirms the importance of balance testing in ecological environments. You et al. found that older adults with MCI exhibited a larger center of pressure deviation (18.1%) compared to older adults without MCI during the dual-task cognitive–walking test [9].

Dynamic postural sway assessment has been determined by qualitative clinical (TUG or BBS) and quantitative postural sway measurements using various sensors (inertial measurement unit sensor-embedded, accelerometer sensor-based postural sway index, or gyro sensor-based tools) and algorithms [10,11]. These measurements have produced relatively reasonable accuracy, specificity, and sensitivity in older adults with a history of falls in laboratory control closed environments [12,13,14,15,16,17,18]. However, there is a lack of research on qualitative clinical (TUG or BBS) and quantitative postural sway measurements in an ecological community environment with higher contextual interference, which could allow for more accurate and discriminative test results than those performed in laboratory environments [19,20,21,22].

A clear need exists to develop an accurate, sensitive, and specific postural sway measurement system to differentiate the falling characteristics of patients with MCI from older adults with a higher risk of falls. We recently developed an innovative wireless wearable postural sway detection (PSD) measurement system using a wearable physiological signal monitoring device (HiCardi, MEZOO Co., Ltd., Wonju, Republic of Korea) designed to measure, analyze, and provide accurate biofeedback on the abnormal postural sway associated with falls during ecologically valid daily activities or environments and which can be incorporated into the TUG and BBS tests. In addition, combining complete 360° tilt sensing with motion-fusion algorithms provides a sensor module that offers precise and dependable orientation estimation, extensive tilt detection, and resilient motion tracking. However, the sensitivity, specificity, and fall prediction accuracy of the PSD measurement system in relation to the standardized BBS and TUG tests have not yet been determined. Therefore, in this study we aimed to (1) validate the fall sensitivity and specificity of PSD by analyzing the area under the curve (AUC) of the receiver operating characteristic (ROC) curve to evaluate the clinical fall classification performance in older adults at a high risk of falls, and (2) predict the fall accuracy in PSD by analyzing the cut-off point in older adults at high risk of falls. We hypothesized that the PSD system would demonstrate acceptable fall sensitivity and specificity in postural sway measurement as well as good accuracy between the extent of postural sway and clinical measures in older individuals at high risk of falls.

## 2. Materials and Methods

### 2.1. Participants

**Figure 1:**
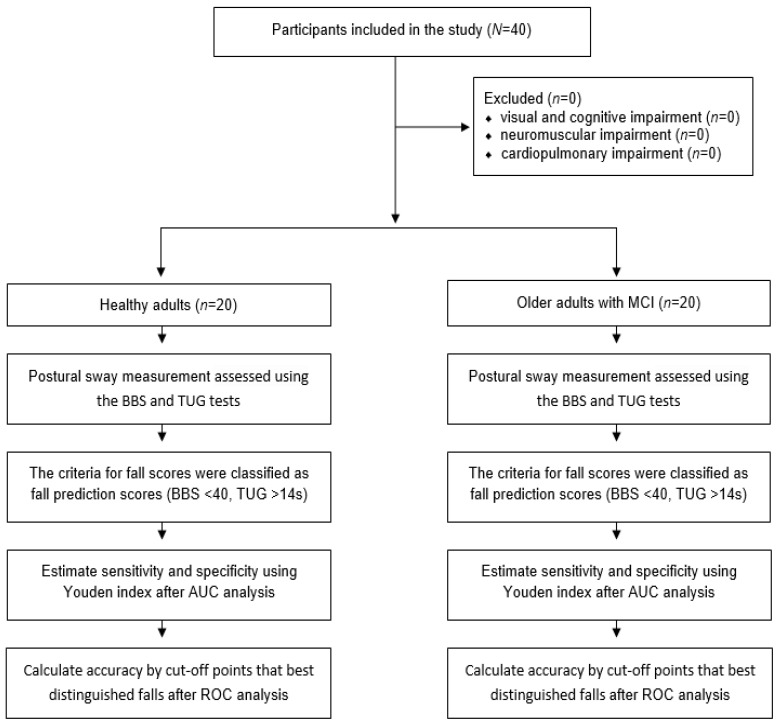
Flow chart.

A convenience sample of 20 healthy adults (mean age, 25.20 ± 3.19 years) and 20 older adults with MCI (mean age, 79.00 ± 8.25 years) were recruited from a major university and senior citizen community center, respectively. Postural sway measurements were conducted in an ecological community rehabilitation center to determine the sensitivity, specificity, and accuracy of the PSD system in healthy adults and older adults with MCI at a high risk of falls. All subjects provided their informed consent for inclusion before they participated in the study. The study was conducted in accordance with the Declaration of Helsinki, and the protocol was approved by the Ethics Committee under no. 1041849-202202-BM-033-02. None of the participants had any known medical issues. The inclusion criteria for older adults with MCI were: (1) an activities-specific balance confidence scale (ABC) score below 1000, (2) age over 65 years, (3) a mini-mental state examination (MMSE) score of 20–23, and (4) a history of falls (e.g., at least one fall in the past 3 months). The exclusion criteria were the presence of visual or cognitive impairment or any neuromuscular or cardiopulmonary impairment influencing the experimental tests [23]. The flow chart is presented in Figure 1.

### 2.2. Description of PSD System

We developed a portable wireless PSD system with nine degrees of freedom. The PSD system consists of a wearable device and monitoring software. The wearable device, the HiCardi (MEZOO Co., Ltd., Wonju-si, Gangwon-do, Republic of Korea), is an 8 g, 42 × 30 × 7 mm, wireless, and wearable adhesive device certified as a medical device for physiological signal monitoring by the Ministry of Food and Drug Safety of Korea. This device can measure single-lead electrocardiograms (ECGs), heart rate (HR), and skin surface temperature, as well as the subject’s motion. The device contains a 32-bit Cortex-M4 processor with Bluetooth Low Energy (BLE) module (NRF52832, Nordic Semiconductor, Trondheim, Norway), an ECG sensor, a temperature sensor, a 50 mAh Li-ion polymer battery, and a 9-axis motion sensor (MPU-9250, InvenSense, San Jose, CA, USA). The motion sensor can measure 3-axis acceleration, 3-axis angular rate, and 3-axis terrestrial magnetism. This device transmits the signals through the BLE module to a laptop equipped with monitoring software. The laptop monitoring software processes the 3-axis acceleration signals received from the wearable device in real time to calculate the 3-axis angles, then displays and stores the 3-axis acceleration signals and angles. The PSD system demonstrated concurrent validity in an intraclass correlation coefficient (${ICC}_{2,k}$) analysis between the sensor and Vernier accelerometer measurements, which showed excellent resemblance among 3-axis posture sway measurements (${ICC}_{2,k}$ = 0.973) [24].

### 2.3. Experimental Procedures

**Figure 2:**
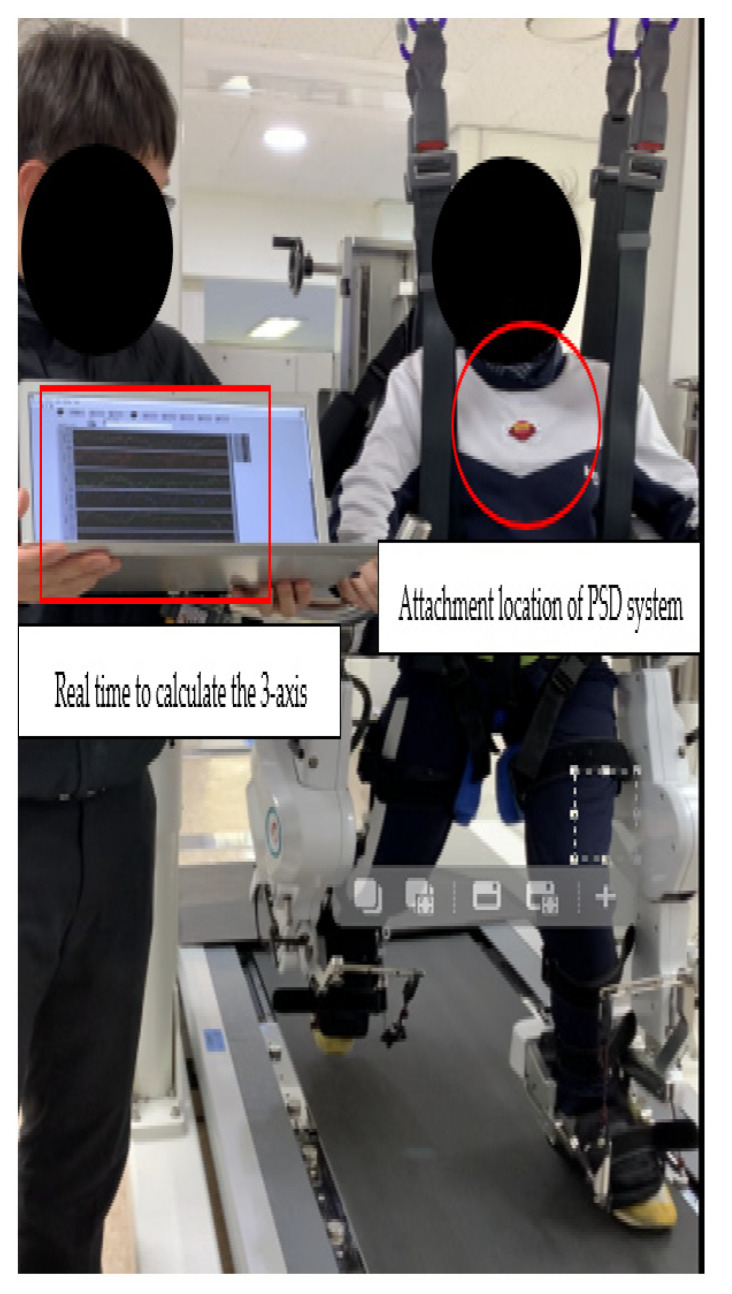
PSD location and real-time signal.

The PSD was constantly attached approximately two-thirds of the way above the xiphoid process and below the jugular notch, and slightly to the left (Figure 2), to collect the controller’s continuously captured data in multiple directions (anterior–posterior (AP), medial-lateral (ML), and vertical (V)) on the postural sway measurements which were recorded simultaneously during clinical tests, including the BBS and TUG tests. The ecological experimental conditions were maintained as consistently as possible by using the same tester and procedure, including providing consistent instructions, calibration, foot positioning, and test sequencing throughout the study. The ecological community rehabilitation center that participated in the study provides rehabilitation and health care for children and adults with disabilities. The risk of falls increases when performed in a substantially compromised or ecological community environment with higher contextual interference (e.g., dual cognitive motor tasks, divided attention tasks) [7,8]. This environment, with its heightened risk of falls, enables accurate and discriminative measurements to predict falls [25].

#### 2.3.1. BBS

The BBS test was used to assess dynamic sitting, standing balance, and the potential risk of falls, and consisted of 14 items, each assigned a score between 0 and 4. The overall score ranged from 0 (“individual with a balance problem”) to 56 (“individual without a balance problem”). A score of 51–56 indicated “no history of falls,” whereas a score between 41–50 represented “a history of falls” with good fall prediction (91% sensitivity, 82% specificity). A score of 40 or less represented a 100% risk of falls [26]. The test–retest reliability of the BBS has been reported to be excellent in older adults (${ICC}_{3,1}$ = 0.97) [27].

#### 2.3.2. TUG

The TUG test was used to evaluate basic mobility and balance in older adults at a high risk of falls [28]. The participants were instructed to rise from an armchair, walk 3 m, return to the chair, and sit down. A time to complete the TUG test of “over 13 s” indicated a 69% probability of being a faller, and “over 14 s” indicated an 83% probability of being a faller [29]. The test–retest reliability of the TUG test is well-established in older individuals (${ICC}_{2,1}$ = 0.97) [30].

#### 2.3.3. ABC

The ABC test was used to evaluate the participants’ self-perceived confidence in maintaining balance and preventing falls. Prior to conducting the experiment, the participants completed a subjective questionnaire; in the case of less-aware participants, the evaluator assisted them in completing the questionnaire. The ABC scale comprises 16 items, each with a rating of 0–100, yielding 1600 total potential points. The participants were required to indicate their level of confidence on a scale of 1–10 for each item. A score below 67% indicated a risk of falling [31].

### 2.4. ROC

In the ROC analysis, PSD was assessed using the AUC to evaluate the fall classification performance [32]. The AUC score indicated the sensitivity and specificity of the fall classification. PSD was analyzed by classifying the measurements of AP, ML, V, and the mean of the three directions, using fall scores as a criterion in clinical balance evaluation tools. The criteria for fall scores were classified as positive (probability of falls occurring) or negative (probability of no falls occurring), which were fall prediction scores (BBS < 40, TUG > 14 s) [26,33]. A score closer to 1 implied a better model and fall classification performance, whereas a score closer to 0.5, which was the minimum AUC score, implied a poor model with no fall classification ability.

### 2.5. Predicting Fall Risk by PSD

To evaluate the fall prediction model for PSD, the sensitivity and specificity of the cut-off points from the ROC curve were analyzed. The Youden’s index in the ROC curve was used to determine the sensitivity, specificity, and accuracy of the cut-off points that best distinguished falls. Among the participants, those who experienced falls within 3 months were considered positive and those who did not were classified as negative, with true positive (TP), false positive (FP), true negative (TN), and false negative (FN) designated as the established cut-off points based on the definitions below. Subsequently, the sensitivity, specificity, and accuracy were calculated [34] based on the following calculations. We defined fall prediction sensitivity, specificity, and accuracy as “0.81 to 0.99 is excellent”, “0.61 to 0.80 is good” and “0.60 to 0.41 is moderate”.

1. True positive (TP) = the number of participants correctly identified as fallers.
2. False positive (FP) = the number of participants incorrectly identified as fallers.
3. True negative (TN) = the number of participants correctly identified as non-fallers.
4. False negative (FN) = the number of participants incorrectly identified as non-fallers.
5. Sensitivity: Sensitivity is the ability to determine cases correctly with respect to falling.
  $$\mathrm{Sensitivity}=\frac{TP}{TP+FN}$$
6. Specificity: Specificity is the ability to determine non-faller cases correctly.
  $$\mathrm{Specificity}=\frac{TN}{TN+FN}$$
7. Accuracy: Accuracy is the ability to correctly differentiate non-faller and faller cases.
  $$\mathrm{Accuracy}=\frac{TP+TN}{TP+TN+FP+FN}$$
8. Youden’s index: The Youden’s index is one of the indicators that assesses the performance of diagnostic tests.
  Youden’s index = MAX (sensitivity + specificity − 1)

### 2.6. The Combination of Complete 360° Tilt Sensing

**Figure 3:**
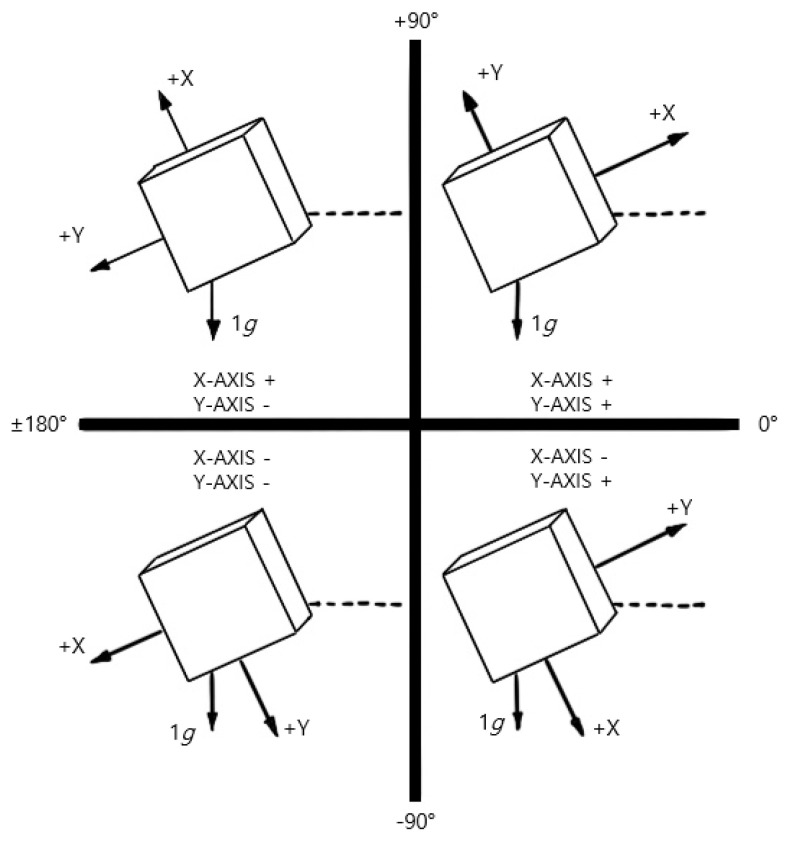
Angle of inclination and sign of acceleration for quadrant detection.

As shown in Figure 3, complete 360° tilt sensing is a technique that combines triple-axis tilt calculations and independent inclination sensing to compute the inclination angles along the X-Y-Z axis. First, the triple-axis tilt calculation was used to calculate the inclination angles in the X-Y plane and the inclination angle between the gravity vector and the *Z*-axis using Equation (3), where X,OUT, Y,OUT, and Z,OUT are the acceleration values measured on the X, Y, and Z axes, respectively. Additionally, *θ*X and *θ*Z represent the respective inclination angles in the X-Z plane. Subsequently, independent inclination sensing was employed to independently compute the inclination angles in the X-Z plane and Y-Z plane using Equations (4) and (5), respectively. Additionally, Equations (4) and (5) can be used to calculate the inclination angles in the X-Z plane and Y-Z plane, which are represented by *θ*X and *θ*Y, respectively. Consequently, the inclination angles along the X-Y-Z axes can be determined by combining the triple-axis tilt calculation and independent inclination sensing.

Equation (1). Triple-axis tilt calculation.

(1) $$\theta =tan^{-1}\left(\frac{A_{X,OUT}}{A_{Y,OUT}}\right)$$

Equation (2). This formula calculates the tilt angle using only the gravitational acceleration value in triple-axis tilt calculations.

(2) $$\varphi =cos^{-1}\left(\frac{A_{Z,OUT}}{\sqrt{A^{2}{}_{X,OUT}+A^{2}{}_{Y,OUT}+A^{2}{}_{Z,OUT}}}\right)$$

Equations (3)–(5). Independent inclination sensing calculations.

(3) $$\theta =tan^{-1}\left(\frac{A_{X,OUT}}{\sqrt{A^{2}{}_{Y,OUT}+A^{2}{}_{Z,OUT}}}\right)$$

(4) $$\psi =tan^{-1}\left(\frac{A_{Y,OUT}}{\sqrt{A^{2}{}_{X,OUT}+A^{2}{}_{Z,OUT}}}\right)$$

(5) $$\varphi =tan^{-1}\left(\frac{\sqrt{A^{2}{}_{X,OUT}+A^{2}{}_{Y,OUT}}}{A^{2}{}_{Z,OUT}}\right)$$

### 2.7. Data Processing

**Figure 4:**
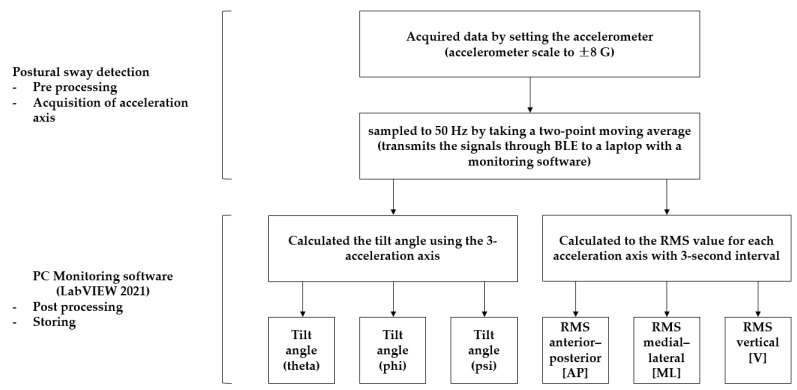
Data processing flow. RMS, root mean square; BLE, Bluetooth Low Energy.

**Figure 5:**
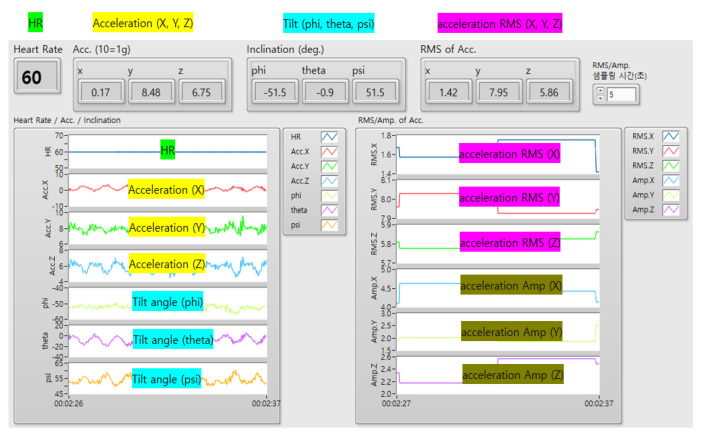
The accelerometer signal data as monitored using LabVIEW 2021 software.

We obtained data by setting the accelerometer scale to ±8 G and monitored the accelerometer signal data using LabVIEW 2021 software at a sampling frequency of 50 Hz. (Figure 4 and Figure 5) The tilt angles (theta, phi, and psi) were calculated by computing the gradient using the three acceleration axes. The magnitude of the acceleration amplitude (AMP) was updated by subtracting the minimum value of the data collected every second from the maximum value. The root mean square (RMS) of the acceleration AMP was updated by collecting data in groups of three. The RMS of the acceleration AMP and tilt AMP data were updated every 3 s and were presented qualitatively in LabVIEW 2021. The AP-, ML-, and V-direction RMS values were used to determine the RMS value for each acceleration axis. The tilt angles (phi, theta, and psi), RMS, and AMP data were stored locally. The tilt angle was calculated every 25 Hz using the arctan formula for the three-axis components of gravitational acceleration (1 G).

### 2.8. Statistical Analysis

Descriptive statistics were expressed as means and standard deviations (SD). The baseline demographic characteristics, clinical balance tests, and PSD data between healthy adults and older individuals with MCI were compared using independent *t*-tests. A power analysis was performed using the G-Power program (version 3.1.9.4; Franz Faul, University of Kiel, Germany) to determine the minimal sample size required based on prior research. Based on previous research, the sample size was determined to be 40 and the power (1 − β = 0.8) was based on the effect size (eta squared, η2 = 0.6). An ROC analysis was used to determine the accuracy of the measurement outcomes, whereas the AUC was used to estimate the sensitivity and specificity in healthy adults and older individuals with MCI. SPSS for Windows (version 26.0, SPSS, Chicago, IL, USA) was used as the software for the statistical analyses. The alpha level was set at 0.05.

## 3. Results

All the participants successfully completed the experimental tests, and their demographic characteristics are presented in Table 1. An independent *t*-test showed that the mean PSD differences between the groups assessed using the BBS and TUG tests were significantly different (*p* = 0.01). The mean difference in the healthy adult group was 0.16 ± 0.20 for BBS and 0.36 ± 0.08 for TUG (*p* < 0.001), whereas in the older individuals with MCI it was 0.41 ± 0.12 for BBS and 0.47 ± 0.12 for TUG (*p* < 0.01).

### 3.1. Fall Prediction AUC

The AUC derived from posture sway data showed a good to excellent fall prediction capability, ranging from 0.82 to 0.94 (Table 2). The BBS was excellent, ranging from 0.91 to 0.94, the TUG was good, ranging from 0.82 to 0.86.

### 3.2. Fall Prediction Sensitivity

The fall sensitivity of the BBS and TUG scores for the PSD measurement data indicates that the sensitivity of the posture sway data was excellent (Table 3). The sensitivities for the AP (BBS = 0.88, TUG = 0.83), ML (BBS = 0.88, TUG = 0.90), and V (BBS = 0.99, TUG = 0.91) directions were excellent. The average value of the three directions was excellent as well (BBS = 0.92, TUG = 0.83).

### 3.3. Fall Prediction Specificity

The fall specificity of the BBS and TUG scores for the PSD measurement data showed good-to-excellent fall specificity for the posture sway data (Table 3). The specificity for the AP direction was moderate-to-excellent (BBS = 0.83, TUG = 0.73), while for the ML direction it was good-to-excellent (BBS = 0.80, TUG = 0.69) and for the V direction it was good (BBS = 0.76, TUG = 0.69). The average value of the three directions was good-to-excellent (BBS = 0.83, TUG = 0.70).

### 3.4. Fall Prediction Accuracy

The fall prediction accuracy of the BBS and TUG clinical measurements for the PSD measurement data showed moderate-to-excellent fall prediction accuracy for the posture sway data (Table 3). The accuracy for the AP direction was good-to-excellent (BBS = 0.81, TUG = 0.53), while for the ML direction was good-to-excellent (BBS = 0.76, TUG = 0.58) and for the V direction it was good (BBS = 0.76, TUG = 0.53). The average value of the three directions was good-to-excellent (BBS = 0.78, TUG = 0.55).

## 4. Discussion

This study investigated the clinical sensitivity, specificity, and accuracy of the PSD system in predicting the fall risk in older individuals with MCI compared with standardized BBS and TUG clinical measurements in an ecological community environment. As hypothesized, the sensitivity, specificity, and accuracy of the PSD system were moderate-to-good, indicating an acceptable ability to discriminate between fallers and non-fallers with established accuracy. Most importantly, the present study provides the first clinical evidence of a novel PSD measurement and demonstrates its sensitivity, specificity, and accuracy in assessing falls in older individuals with MCI.

The AUC analysis showed excellent sensitivity (0.82–0.99) in predicting fallers among older adults with MCI when the PSD measurements of the AP, ML, and V directions were selectively compared with clinical BBS and TUG measurements in an ecological community environment. Specifically, a greater sensitivity was observed in the V direction, as demonstrated by the sensitivity for the BBS (0.99) and TUG (0.91) tests across all participants (Table 3). Our findings differ from those of earlier studies that examined postural sway measurements in older individuals [35,36]. Ghahramani et al. reported an AUC sensitivity value of 0.75 in detecting falls from a postural sway measurement in 86 older individuals with a history of falls using accelerometer sensors and customized algorithms [35]. Similarly, Kelly et al. calculated an AUC sensitivity value of 0.61 for predicting falls among 712 older individuals with a history of falls when employing accelerometer sensors and the random forest algorithm [36]. A possible rationale for this difference in sensitivity may be due to the different types of sensors and algorithms used. Specifically, unlike the methods used in previous studies [35,36], our PSD system is equipped with a more sophisticated 3-axis gyroscope, accelerometer, and magnetometer and an onboard Digital Motion Processor™ with a special algorithm capable of processing complex motion-fusion or artifacts [37].

The AUC specificity analysis showed moderate-to-excellent specificity (0.69–0.90) for predicting fallers in among older adults with MCI when the PSD measurements in the AP, ML, and V directions were selectively correlated with clinical BBS and TUG measurements in an ecological community environment. Our findings are compatible with those of recent studies that examined the postural sway measurement specificity in older adults [38,39]. Tulipani et al. reported a relatively excellent specificity of 0.90 in detecting falls among 36 older individuals with multiple sclerosis using accelerometer-derived metrics to classify the fall status during the unsupervised 30 s chair stand test [38]. Bet et al. showed a specificity of 0.76 when correlating with the TUG test using a single accelerometer on the waist near the center of mass along with associated postural sway accelerometry data in an analysis of 74 older individuals without a recent history of falls [39]. The reasonable predictability for discerning the presence or absence of fall risk in older adults may be related to the PSD system’s ability to accurately estimate the orientation of the module in a three-dimensional space as well as the special “smart” algorithm used to automatically detect and filter out sensor bias and motion artifact noise. Thus, the system can minimize errors and identify dynamic postural sway or deviation frequency data, which are necessary for estimating the presence or absence of fall-related postural sway.

The ROC accuracy analysis showed moderate-to-good accuracy (0.53–0.81) in predicting fallers among older adults with MCI using PSD dynamic postural sway measurement of the AP, ML, and V directions when compared with the clinical measurements from BBS and TUG tests in an ecological community environment. The PSD system is an accurate measurement tool that can distinguish between falls in older individuals with MCI and falls in healthy adults. The accuracy of PSD measurement was comparable to that reported in previous studies [40,41]. Howcroft et al. reported a relatively moderate accuracy of 0.54 using a prediction algorithm model programmed in wearable accelerometer sensors to predict falls in older adults [40]. In contrast, Delgado-Escano et al. observed good accuracy (0.79) in fall-related postural sway detection and identification using deep learning methods in 15 older and 57 healthy adults [41]. A possible explanation for this difference is that the PSD system’s on-board calibration function calibrates the output data of the PSD sensor to minimize errors and improve the reliability and accuracy of the sensor by up to ten-fold. On-board calibration uses zero offset and scale factor calibration to calculate and correct errors in the accelerometer [42,43,44]. Notably, the fall accuracy was consistently high in the BBS test, supporting the evidence that the BBS test evaluates various balance-related items and shows high validity and reliability [45]. In fact, Park et al. evaluated the accuracy of the BBS test in detecting falls in older individuals and found that the BBS test had a sensitivity of 0.84 for predicting falls [46].

This study has a number of limitations. One main limitation is that the present PSD measurements involved relatively quick clinical measurements compared to the clinically standardized BBS and TUG tests. However, daily life logs and unsupervised data measurements in an individual’s environment can provide ecologically valid postural sway data in the real world. Another limitation is that our results cannot be generalized to other diverse demographic groups or clinical contexts. Hence, careful interpretation should be made when applying the current findings, warranting future research in different pathological populations with dynamic balance disorders and movement test conditions.

## 5. Conclusions

The present study established the sensitivity, specificity, and accuracy of the PSD system in predicting falls compared with those of the BBS and TUG tests. Our novel PSD system is a useful assessment tool for the accurate evaluation of dynamic balance and prediction of falls in older individuals with MCI at a high risk of falls. The PSD system measurement can provide an alternative source of clinical evidence when designing fall assessments, effective or sustainable preventions, and interventional programs for older individuals with a high risk of falls using the normative and fall detection data established in the present study.

## Figures and Tables

**Table 1:** Demographic characteristics of the participants (*N* = 40).

Variable		Healthy Adults (*n* = 20)	Older Adults with MCI (*n* = 20)	*p*-Value
Age (years), mean ± SD		25.20 ± 3.19	79.00 ± 8.25	0.001
Height (cm), mean ± SD		169.65 ± 9.37	157.25 ± 8.45	0.001
Weight (kg), mean ± SD		67.70 ± 13.83	59.40 ± 7.30	0.024
Male (%)		50	50	-
Female (%)		50	50	-
BBS	Clinical tests (score) mean ± SD	54.61 ± 1.33	39 ± 5.88	0.001
	Posture sway data * mean ± SD	0.16 ± 0.20	0.41 ± 0.12	0.001
TUG	Clinical tests (second) mean ± SD	9.42 ± 0.25	14.11 ± 1.37	0.001
	Posture sway data * mean ± SD	0.36 ± 0.08	0.47 ± 0.12	0.01

BBS, Berg Balance Scale; TUG, Timed Up and Go; SD, Standard deviation; * Mean of three directions.

**Table 2:** AUCs, standard errors, confidence intervals, and *p*-values in the BBS and TUG tests.

Subscales		AUC	SE	95% Confidence Interval		*p*-Value
				Lower	Upper	
BBS	AP	0.91	0.50	0.82	0.99	0.001
	ML	0.94	0.40	0.87	0.99	0.001
	V	0.91	0.50	0.81	0.99	0.001
	Mean *	0.92	0.47	0.84	0.99	0.001
TUG	AP	0.83	0.68	0.69	0.96	0.002
	ML	0.82	0.73	0.68	0.96	0.002
	V	0.86	0.62	0.74	0.98	0.001
	Mean *	0.84	0.68	0.70	0.97	0.002

AUC, Area under the curve; SE, Standard error; BBS, Berg Balance Scale; TUG, Timed Up and Go; AP, Anterior—posterior; ML, Medial—lateral; V, Vertical; Mean *, Mean of three directions.

**Table 3:** Cut-off point, sensitivity, specificity, and accuracy of each subscale.

Subscales		Cut-Off Point	Sensitivity	Specificity	Accuracy
BBS	AP	0.39	0.88	0.83	0.81
	ML	0.34	0.88	0.90	0.76
	V	0.34	0.99	0.76	0.76
	Mean *	0.36	0.92	0.83	0.78
TUG	AP	0.50	0.82	0.73	0.53
	ML	0.39	0.91	0.69	0.58
	V	0.49	0.91	0.69	0.53
	Mean *	0.46	0.88	0.70	0.55

BBS, Berg Balance Scale; TUG, Timed Up and Go; AP, Anterior—posterior; ML, Medial—lateral; V, Vertical; Mean *, Mean of 3 directions.

## Data Availability

All data are available upon request.
